# Medical and Philosophical News

**Published:** 1781-09

**Authors:** 


### SECTION III.

### Medical and Philosophical News.

THE Academy of Belles Lettres, Sciences, and Arts, at Marseilles, have proposed the following subject for a prize medal of three hundred livres value: " The plant vulgarly called *barbe* " *de Renard*, known to botanists by the name of " *Tracanta Massiliensis*, which grows naturally " on the borders of the Mediterranean, is it the " same as that which is cultivated in the Levant " for the extraction of Gum Tragacanth; and, " if so, what would be the best method of cul- " tivating it for that purpose in Provence ? "— The dissertations are to be sent to M. Mouraille, secretary to the academy, before the 1st of Jan. 1783.

---

Extract of a letter from Dr. Saury, physician to his imperial and royal majesty, dated Mosul in Asia, Dec. 7, 1780.

" The Hydrophobia is unknown in the island of Cyprus, at Sidon, at Tripoli in Syria, along the whole coast of Syria, and even at Aleppo. The Europeans who inhabit those countries all agree, that dogs never go mad there. Among the the caufes of canine madnefs, Boerhaave enumerates a burning climate, a hot dry feafon, want of water, and the feeding on putrid flefh. All thefe caufes prevail in Cyprus, along the coaft of Syria and at Aleppo. In Cyprus the climate is very hot, and water extremely fcarce. There is fo little rain, that from Eafter to November the gardens lie uncultivated for want of water. Every body knows, that the coaft of Syria is equally hot, and the air very dry. On the other hand, in all the towns, under the Mahometan government, dogs are very numerous, and as they are no particular perfon's property they live on the offal of the fhambles and what they can pick up in the ftreets. In the country they are deftitute of water, and feed upon dead camels, horfes, &c. It would feem then, that the want of water, and the other caufes I have mentioned, are not fufficient to excite madnefs in an animal, and that there is fome other caufe, as yet unknown, which, in Europe, renders this diforder fo frequent. It is very rare at Moful, neverthelefs I have this year feen a mad dog here who bit a Turk. The Dominicans, who are the only European phyficians at this place, recommended local fcarifications, and the bite produced no bad effect."

Extract

Extract of a letter from Dr. Olaus Acrell, phyſician at Upſal.

"Both the cauſe and the cure of the Tinea Capitis are as yet far from being ſatisfactorily aſcertained. In Sweden women very often ſucceed in the removal of this complaint by extracting the hairs by their roots, and afterwards waſhing the head repeatedly with a weak alkaline lixivium, or a ſolution of vitriol or alum. This proceſs however is tedious, ſometimes requiring many months, and even repeated extraction of the hair before the cure is completed. In my own practice, I have generally found the following method ſucceed the beſt: I firſt draw out the hair and then waſh the head with a decoction of centaureum minus, in which I diſſolve a ſmall portion of corroſive ſublimate. At the ſame time I give internally either the æthiops antimonialis or a table ſpoonful of the following mixture every morning and evening: ℞. Merc. corroſ. ſublim. gr. vj. aq. diſtill. ℥ix. aq. cinnamom. ſimp. ℥ij. ſpir. vin. rectif. ſyrup. alth. an. ℥j. M.

---

In the *Journal de Medecine* for July 1781, M. Chartier, phyſician at Anjou, relates the caſe of a man a man forty years of age, who was attacked with a deep-feated, pungent pain in the right fide, extending from the fourth true rib to the firft falfe rib, and attended with a dry cough and difficulty of breathing. Thefe fymptoms continued for three or four months, during which time he was unable to lie on his right fide. After that he began to expectorate a whitifh frothy mucus. His pulfe was but little affected, yet his countenance acquired a yellowifh tinge, and in a fhort time he died. Upon diffection the right lobe of the lungs was found adhering to the pleura. The left was totally fuppurated. The pus had eroded the pericardium, and deftroyed more than two-thirds of its fubftance. The liver, though in other refpects apparently found, was of an extraordinary bulk, extending from the right to the left hypochondrium. The fpleen was likewife much enlarged, the ftomach, by the preffure of the two vifcera, was forced down into the umbilical region, and the omentum into the epigaftric region.

In the fame work M. Maurice, furgeon at Chinon, gives an account of a young man aged twenty-two years, who had his left *tendo Achillis* cut through by a fickle. The wound was tranfverfe, deep, and two inches and a half long.

 The

The muſcles of the calf of the leg were contracted under the ham, that part of the tendon next to the muſcles being drawn at leaſt three inches upwards, while the lower end of it projected conſiderably. In fifty-five days, with the aſſiſtance of ſuitable bandages, the two portions were reunited, and the patient was able to walk.

---

Signor Bartollozi, an Italian naturaliſt, has obſerved, that the fecundation of plants does not always depend on a ſeminal duſt. In the *aſclepias* this proceſs is effected by means of a tranſparent glutinous liquor, a drop of which is to be ſeen at the top of the filament, the anthera containing no duſt.——*Journ. de Phyſique*, for April 1781.

---

M. Boué, ſurgeon at Meulan ſur Seine, has publiſhed an account of a woman who was adviſed to make uſe of a warm bath impregnated with a large quantity of quicklime, as a cure for the rheumatiſm. She uſed it twice, and remained in it an hour each time. The effects of this improper application were a total loſs of hair and cuticle from every part that came in contact with the the water, and an apparently incurable contraction of all her limbs.——*Gazette de Santé.*

---

The dysentery which was so prevalent in London during the autumns of 1779 and 1780 seems to be equally rife at present. Some few patients were attacked with it so early as the last week in July; but it became much more general towards the latter end of August and the beginning of September. In the greater number of cases it has been attended with profuse evacuations by stool of a watery, mucous matter mixed with blood; severe gripings of the bowels, and pain in the back and loins, with tenesmus, nausea, and fever: but some have had violent *tormina*, with little or no discharge by stool. Some few old and infirm persons have sunk under it, and we have seen one instance where it proved fatal by coming on in a case of confluent small pox about the time of the crisis; but in general it has yielded to a proper mode of treatment. Opium, after the necessary evacuations have been premised, has proved of great use. In some cases where the tormina were extremely severe we have found it necessary to allay the irritation in the bowels by giving opium even at the beginning, joined with some purgative medicine. Bleeding, when the pain of the bowels has been accompanied with a high pulse and other inflammatory symptoms, has generally had a good effect. When the sickness at the stomach has been considerable, an emetic has commonly proved efficacious. In several cases where visceral obstructions have been brought on by cordials, astringents, and other improper remedies, we have experienced good effects from small doses of calomel with a grain of opium, given occasionally at bed-time.

---

An English translation of Professor Bergman's Essay on the application of chemistry to the various purposes of life, is now in the press in London, with the addition of some useful chemical tables by the translator.

